# Alpha-Fetoprotein Binding Mucin and Scavenger Receptors: An Available Bio-Target for Treating Cancer

**DOI:** 10.3389/fonc.2021.625936

**Published:** 2021-02-25

**Authors:** Bo Lin, Qiujiao Wang, Kun Liu, Xu Dong, Mingyue Zhu, Mengsen Li

**Affiliations:** ^1^ Hainan Provincial Key Laboratory of Carcinogenesis and Intervention, Hainan Medical College, Haikou, China; ^2^ Institution of Tumor, Hainan Medical College, Haikou, China

**Keywords:** alpha-fetoprotein receptors, structure, function, mucin receptors, scavenger receptors

## Abstract

Alpha-fetoprotein (AFP) entrance into cancer cells is mediated by AFP receptors (AFPRs) and exerts malignant effects. Therefore, understanding the structure of AFPRs will facilitate the development of rational approaches for vaccine design, drug delivery, antagonizing immune suppression and diagnostic imaging to treat cancer effectively. Throughout the last three decades, the identification of universal receptors for AFP has failed due to their complex carbohydrate polymer structures. Here, we focused on the two types of binding proteins or receptors that may serve as AFPRs, namely, the A) mucin receptors family, and B) the scavenger family. We presented an informative review with detailed descriptions of the signal transduction, cross-talk, and interplay of various transcription factors which highlight the downstream events following AFP binding to mucin or scavenger receptors. We mainly explored the underlying mechanisms involved mucin or scavenger receptors that interact with AFP, provide more evidence to support these receptors as tumor AFPRs, and establish a theoretical basis for targeting therapy of cancer.

## Introduction

Alpha-fetoprotein (AFP) plays an important role in inducing malignant transformation of cancer cells, regulating cell proliferation, migration, apoptosis and immune escape, and is used as clinical biomarker to diagnose hepatocellular carcinoma (1–5). In the 2018 EASL clinical practice guidelines, AFP can be used as an indicator for the diagnosis and prognosis of advanced HCC (6), and high serum AFP may indicate drug resistance and a poor prognosis in many HCC patients (3–6). The malignant behavior of AFP is mainly mediated by its receptors (AFPRs), which are present on the cell surface and their expression in cells is associated with presence of AFP (7–9). These findings implicated that during the development and the carcinogenesis, the expression of AFPRs is not only accompanied by the expression of AFP, but also can mediate the extracellular AFP endocytosis which plays a role in inducing the growth and differentiation of the cells promoting their carcinogenesis.

## The Role of Classic AFP Receptors and AFP

**Figure 1:**
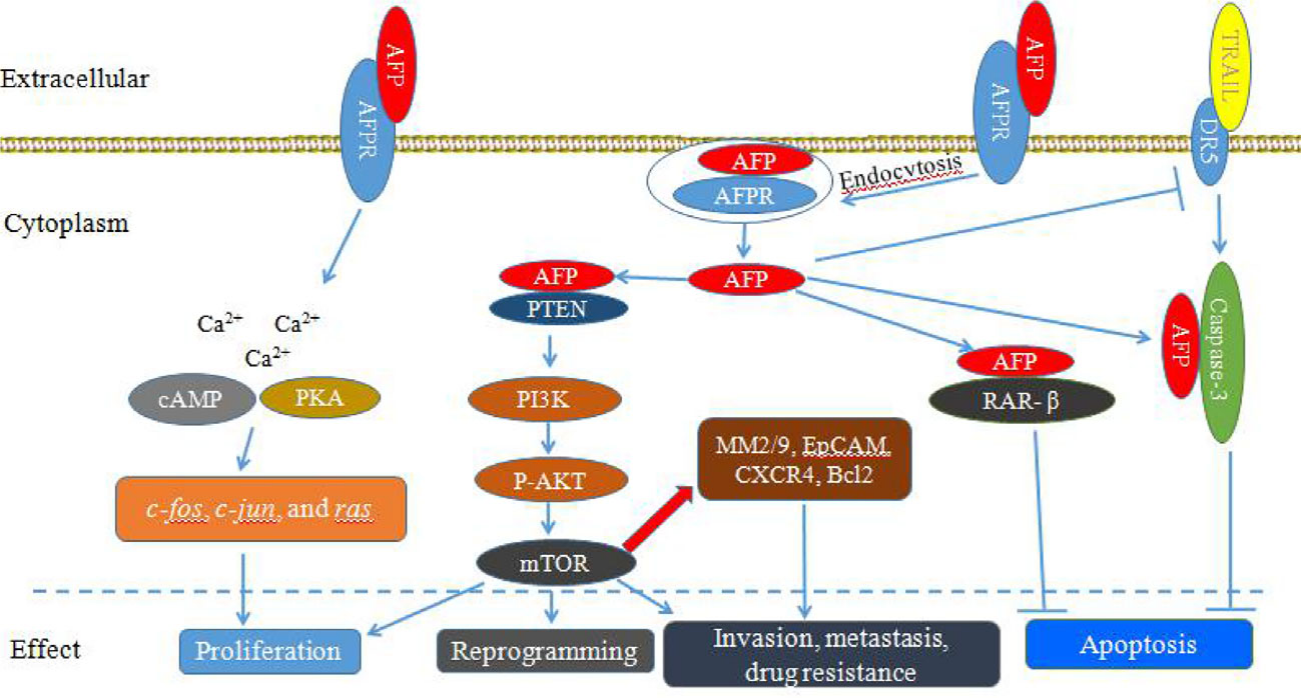
Schematic diagram of Alpha-fetoprotein (AFP) bound to AFP receptors (AFPRs) promoting the malignant transformation of liver cancer cells. AFP binds to AFPRs and undergoes receptor-mediated endocytosis. Then, AFPRs and intracellular AFP activate signal transduction pathways and mediate the proliferation, invasion, metastasis, reprogramming and antiapoptotic responses of liver cancer cells.

AFP can bind to AFPRs and activate the cyclic adenosine 3’, 5’-monophosphate (cAMP)-protein kinase A (PKA) pathway and induce Ca^2+^ influx, which increases intracellular cAMP and PKA, enhances DNA synthesis, promotes the expression of the oncogenes *c-fos*, *c-jun*, and *ras*, and stimulates the growth of liver cancer cells (**Figure 1**) (10–12). Additionally, after binding with AFPRs, it lead to triggering growth-promoting signals and promoting the endocytosis of AFP. The endocytosed AFP (we also call cytoplasmic AFP or AFP briefly) then binds with certain cytoplasmic proteins, leading to the activation or inhibition of signaling pathways. The endocytosed AFP can interact with PTEN and activate the PI3K/AKT/mTOR pathway, which promotes the malignant behavior of hepatocellular carcinoma (HCC) cells by upregulating the protein expression of mTOR (13–17) (**Figure 1**).

AFP not only can play an important role in regulating the proliferation of tumors, but also can enhance the anti-apoptotic effect of cancer cells (18–20). Cytoplasmic AFP can enhance resistance to apoptotic factors by affecting the TGF-β and p53/Bax/caspase-3 signaling pathways (18, 21). AFP can compete with all-trans retinoic acid (ATRA) to bind retinoic acid receptor-β (RAR-β) leading to promoting the expression of Bcl-2 and inhibiting apoptosis (22–25). In addition, AFP can inhibit the expression of death receptor 3 (DR3), and bind with caspase-3, resulting in inhibition of apoptosis of tumor cells induced by tumor necrosis factor-related apoptosis-inducing ligand (TRAIL) (**Figure 1**) (22–25). AFP can also promote HCC cell invasion and metastasis through upregulating the expression of matrix metalloproteinase (MMP) 2/9 (**Figure 1**), epithelial cell adhesion molecule (EpCAM), and C-X-C chemokine receptor type 4 (CXCR4), and AFP knockout significantly inhibits the migration and invasion abilities of HCC cells (26–28).

Since many tumor cells express AFPRs and these cells can take up AFP and exert malignant effects, the elucidation of AFPRs structure and function will help expand academic understanding of cancer cell transformation, proliferation, progression, migration and invasion. This understanding will help specialty design drugs to selectively eliminate cancer cells or design vaccines to treat cancer (7–9). The search for the structure for AFPRs in tumor cells has long endured for three decades (9, 29, 30), however, due to the complex polymer structure and carbohydrate composition, a complete elucidation of AFPRs have failed despite several published attempts.

AFPRs were firstly found to bind to AFP in the pits of the membrane bilayers and subsequently packaged in endosomal vesicles and vesicular bodies; that were endocytosed by the cells and then transported to the Golgi network. Finally, the vesicles released AFP and AFPRs, which were distributed in the cytoplasm and the nucleus, and participated in signal transduction pathways, recycling or lysosomal degradation (31, 32). Subsequent studies found that the uptake AFP in the pits of different cell membrane by various receptors were identified as scavenger family members that included mannose receptor (CD206), CD36, LOX1, SRA1, and SRB1 (29, 33, 34). Additionally, Laderoute successfully isolated and partially identified other AFP receptors on epithelial tumor cells that exhibited peanut agglutinin (PNA) lectin reactivity in 1994 (35). These heavily glycosylated proteins have also been detected on human adenocarcinoma cells, macrophages, dendritic cells, thymocytes, and leukemia cells, which also express PNA-responsive cell surface proteins (30). Reports of PNA-reactive glycoproteins on tumor cells often involve the growth and progression of those cells (30). In a later study, these AFP tumor receptors were proposed as mucin receptors which display molecular weights of 61 and 65 kD similar to the PNA reactive receptors (9, 30, 34).

Recent studies showed that there does not exist just one universal AFPR and supported the presence of multiple AFP cell surface receptors which may also include: a) chemokine receptors (34, 36–39), b) cation channel proteins (34, 40–44), c) cell cycle-associated proteins, d) extra-cellular matrix proteins, e) G-coupled lysolipid receptors, f) intra-cytoplasmic proteins, and g) serum IgM binding (34). Although many diverse binding proteins or receptors for AFP have been reported, the complete elucidation of AFPRs is still elusive. Here, we focused on the two types of binding proteins or receptors, namely, A) the mucin (MUC) family, and B) the scavenger receptor (SR) family which may serve as AFPRs that have been reported in the biomedical literature (9, 29, 30, 34). We mainly discussed in detail the events of mucin or scavenger receptors binding to AFP that involve AFP uptake, cytoplasmic trafficking, signal transduction and molecular cross-talk, which affect cell functions and fate and promote the malignant transformation of tumors, and provide further evidence in support of them as tumor AFPRs.

## Mucin Receptors

Based on structure and localization, mucins can be classified into two types: A) secreted and B) transmembrane mucins (mucin receptors). Transmembrane mucins contain a hydrophobic membrane spanning(transmembrane) domain, and they play roles in regulating various signaling pathways (45). There are the MUC1 to MUC22 mucin family members which currently have been identified in humans; that include MUC1, MUC3A, MUC3B, MUC4, MUC12, MUC13, MUC14, MUC15, MUC16, MUC17, MUC20, MUC21, and MUC22 which are belong to the transmembrane mucins. The extracellular domain is mainly composed of a variable number of tandem-repeat (TR) domains, SEA (sea urchin sperm protein, enterokinase, and agrin) domains, epidermal growth factor (EGF)-like domains and an amino terminal extracellular region that has densely populated with glycans. The transmembrane domain anchors the protein with a single membrane-spanning domain incorporated into the plasma membrane and extending to a carboxy terminal intracellular cytoplasmic tail (45).

**Figure 2:**
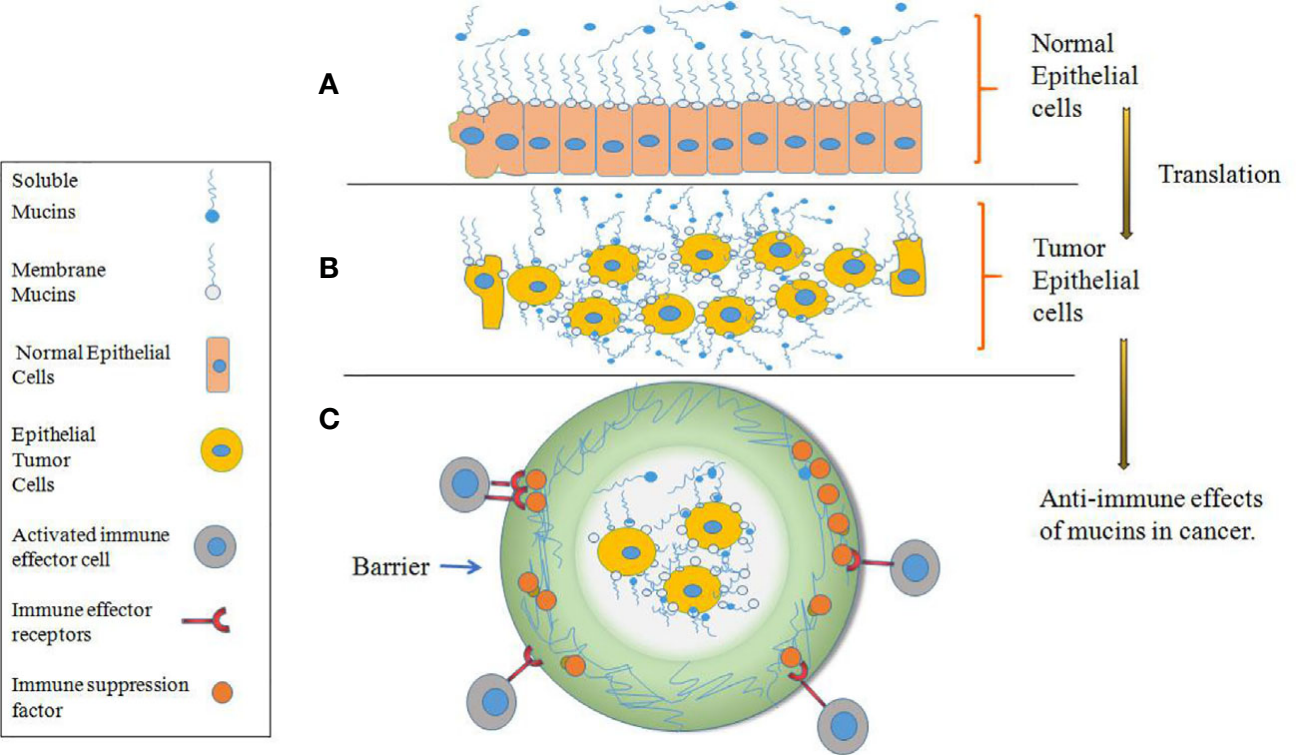
Expression of mucins on normal versus tumor epithelial cells. **(A)** Mucins are expressed at the surface of densely packed columnar cells in normal epithelium which form of protective and lubricating function on the mucosal cell layer. **(B)** After normal epithelial cells become transformed into malignant tumor cells, mucins are highly expressed on the transformed tumor cells and cover all surface areas of the cell membrane, and the tumor cells become rounded in shape, increase cell-cell porosity and lose polarity. **(C)** In tumor epithelial cells, mucins provide an impenetrable barrier for immune effector cells and prevent an antitumor response.

Under physiological conditions, mucins are primarily expressed and anchored within the membrane surface of the mucosa of epithelial cells and other cells, and some mucins are subsequently cleaved by proteolytic enzymes and enter the extracellular fluid and serum (46, 47). The main function of mucins is to form a protective layer over organs, provide lubrication, mediate cell signal transduction and create a physical barrier against pathogens, but this proportion may disappear during tumor transformation and development (48–52). As shown in **Figure 2A**, mucins are expressed on the outer surface of normal cells, and the cells beneath the mucosal layer are columnar and densely packed. However, after normal epithelial cells become transformed into malignant tumor cells, they increase their expression and accumulation of mucins on the tumor cell surface. The tumor cells become rounded in shape, increase cell-cell porosity, lose polarity and lose adhesion connections between adjacent cells (**Figure 2B**) (30, 50–52). In tumor cells, Mucins also might affect the immune response in several ways. They might provide an impenetrable barrier for activated immune effector cells, so preventing an antitumor response; they might suppress a tumor immune response through receptor–ligand interactions. For example, they might accelerate the expression of immunosuppression factor to bind immune effector receptors and inactivate immune effector cells (**Figure 2C**) (50–52).

Aberrant mucin expression can contribute to the loss of epithelial cell polarity and promote epithelial-mesenchymal transition (EMT), which leads to enhanced cell motility and invasion and is a key step in tumorigenesis, thus, mucins are considered to be diagnostic-prognostic markers, as well as therapeutic targets in many cancers (50–60). In the transmembrane mucin family, MUC1 is considered to be an “oncofetal” antigen in the hepatobiliary system and plays a key role as an oncogene in the development of human liver tumors (61–68). MUC1 is not only related to human hepatic tumorigenesis but also has some characteristics of AFPRs (9, 30, 34). The overexpression of the oncoprotein MUC1 correlates with the aggressiveness of malignant tumors and the poor survival of cancer patients and is associated with approximately 80% of human cancers (9, 30, 45). High expression of MUC1 has been linked to cancer cell proliferation, invasion, apoptosis, and transcription, and the downregulation of MUC1 in cancer cells inhibits cell migration and metastasis (9, 61, 69, 70). In addition, the cytoplasmic domain of MUC1 (MUC1C) has several phosphorylation sites, and may be activated by AFP to interact with signaling molecules to inhibit apoptosis and regulate the transcription of several genes to promote proliferation, ultimately converting the cell into a malignant phenotype (61, 69–71). These functions of MUC1 are similar to those of the AFPR (9, 30, 69–71), and so MUC1 might serve as a receptor for AFP.

**Figure 3:**
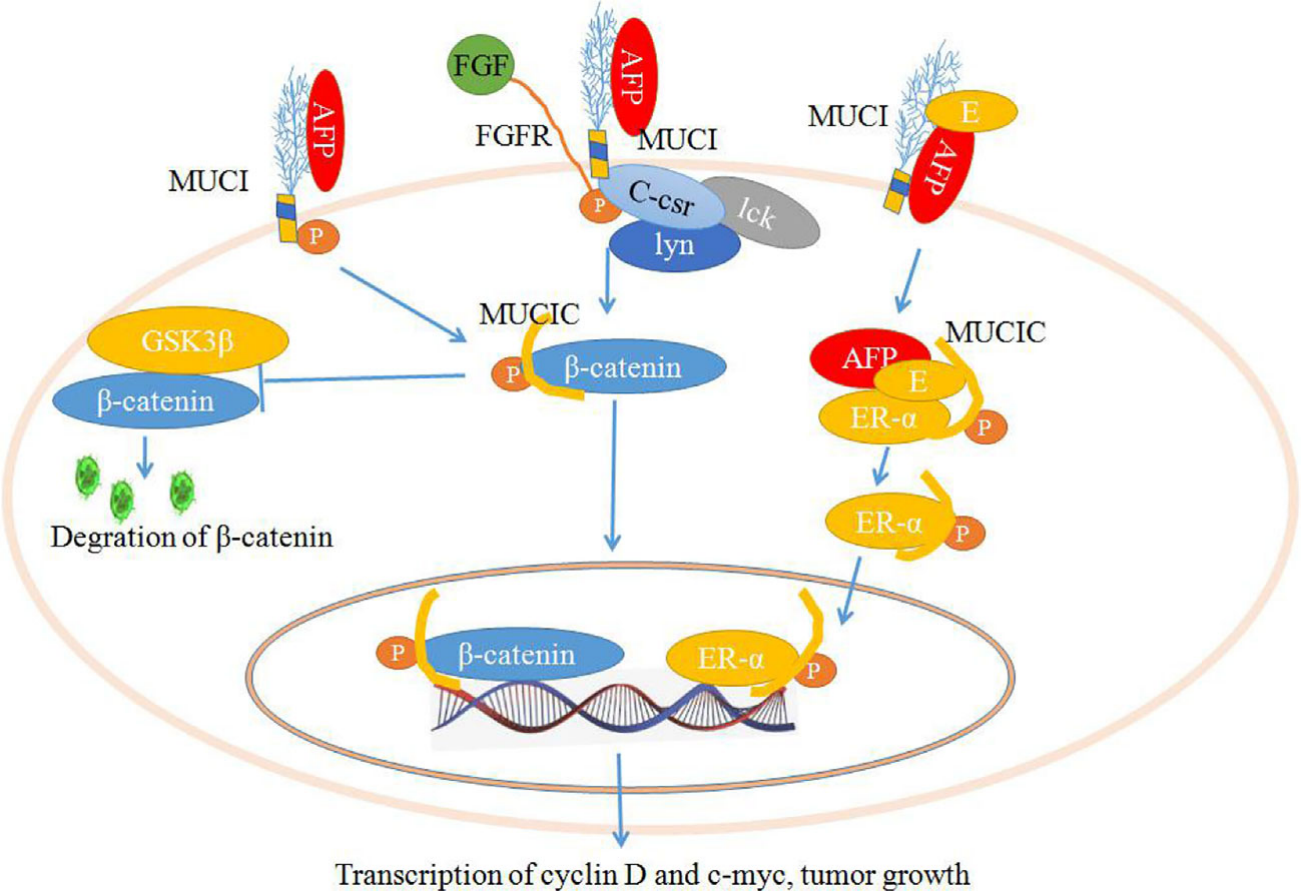
Schematic diagram of the mechanisms by which mucin 1 (MUC1) promotes cell proliferation. Alpha-fetoprotein (AFP) or fibroblast growth factors (FGFs) bind to MUC1 and induce phosphorylation of the cytoplasmic domain of MUC1 (MUC1C), which enhances MUC1C interactions with β-catenin and further increases β-catenin levels in the cytoplasm and nucleus by inhibiting GSK3β-mediated degradation. Furthermore, β-catenin contributes to cell proliferation by increasing the expression of cell cycle progression genes such as cyclin D and c-myc. AFP may also interact with estrogen and MUC1, which may induce the endocytosis of AFP and estrogen and result in the promotion of MUC1C and ERα (estrogen receptor α) localization to the nucleus and increase the expression of cell cycle progression genes, further promoting the growth of malignant cells.

As shown in **Figure 3**, the stimulation of fibroblast growth factors (FGF) or AFP may induce the phosphorylation of MUC1C, which enhances the binding affinity of β-catenin. Ultimately, MUC1C promotes β-catenin localization to the nucleus (45, 71). Furthermore, nuclear β-catenin may contribute to cell proliferation by increasing the expression of cell cycle progression genes such as cyclin D and c-myc. Additionally, MUC1C interacts with β-catenin and further increases β-catenin levels in the cytoplasm and nucleus by inhibiting GSK3β-mediated degradation of β-catenin. The members of the src family (c-Src, Lyn, and Lck) have also been shown to induce the phosphorylation of MUC1C, which may contribute to the docking of β-catenin, increasing β-catenin levels to promote cell proliferation (45, 71, 72). AFP may also interact with estrogen and MUC1, which may induce the endocytosis of AFP and estrogen into cells. Then, the endocytosis of AFP and estrogen recruits MUC1C and ERα (estrogen receptor α) and promotes nuclear localization of these factors. Aberrant localization of MUC1C and ERα in the nucleus also increases the expression of cell cycle progression genes, which further promotes the growth of malignant cells (**Figure 3**) (45, 73, 74).

**Figure 4:**
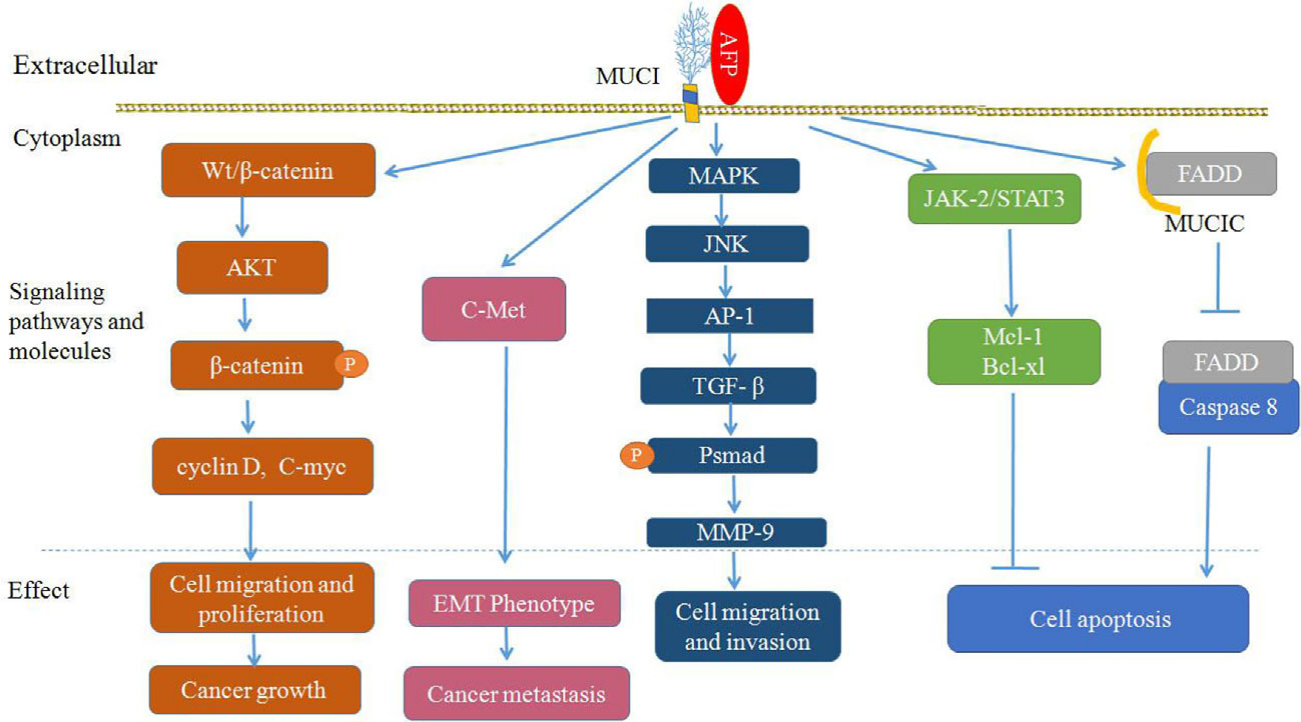
Proposed model of mucin 1 (MUC1)-mediated promotion of hepatocellular carcinoma (HCC) growth, tumor progression and metastasis. Alpha-fetoprotein (AFP) may bind to MUC1 and activate Wnt/β-catenin, mitogen-activated protein kinase (MAPK) with JNK/AP-1 and JAK2/STAT3, which are known to stimulate cancer cells proliferation, survival, migration and invasion, and inhibit apoptosis of cancer cells.

MUC1 not only regulates Wnt/β-catenin signaling pathways, but also influences HGF/c-Met, TGF-β, MAPK with JNK/AP-1, and JAK2/STAT3 signaling pathways (**Figure 4**) (62–67, 74, 75). MUC1 can also interact with c-Met and participate in the EMT process during hepatocarcinogenesis (62). MUC1 can promote the migration and invasion of HCC by increasing TGF-β levels *via* the MAPK/JNK/AP-1 signaling pathway (65, 66), and MUC1 can also promote TGF-β-related signaling molecules (Smads) or elevate other transcription factors, such as MMP-9, which supports the stimulating influence of MUC1 on the proliferation, migration and invasiveness of HCC cells (64, 74). Additionally, it has been shown that MUC1 can mediate protection against irradiation-induced apoptosis, which was associated with the activation of the JAK2/STAT3 signaling pathway and the induction of the antiapoptotic proteins Mcl-1 and Bcl-xL in mitochondria (75), and MUC1C can also inhibit the extrinsic apoptotic pathway by direct interactions with FADD and block FADD-mediated recruitment of caspase-8 (45, 76) (**Figure 4**).

**Figure 5:**
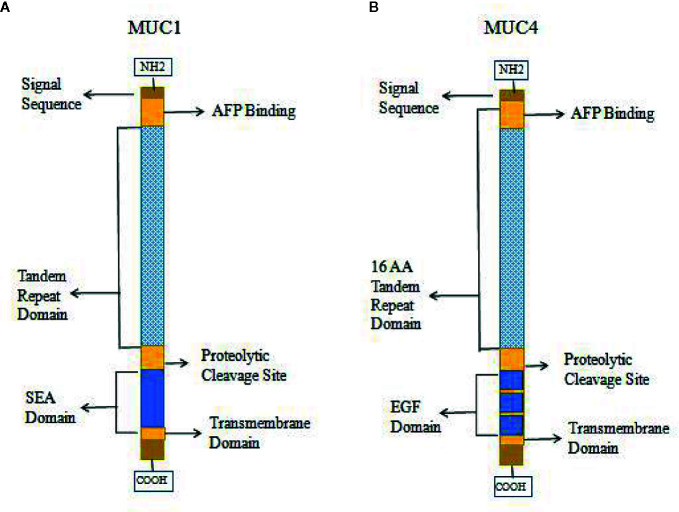
Diagrams of the domains of mucin 1 (MUC1) **(A)** and MUC4 **(B)**.

High expression of MUC1 has been linked to cancer cell proliferation, invasion, metastasis, and apoptosis inhibition. This function of MUC1 is similar to that of AFPR, and it is possible that MUC1 can bind and be activated by AFP, which leads to phosphorylation of the cytoplasmic tail of MUC1 and the promotion of AFP endocytosis to activate related pathways (9, 30, 34, 45, 74). Therefore, MUC1 may be harbor a function as AFPR in tumor cells. Studies also showed that other mucin receptors such as MUC4 may be proposed as AFP cell surface receptors (9, 30, 34). AFP could be a decoy ligand for MUC1 and MUC4, and it could bind and occupy the mucin receptor binding pocket (9, 30, 34, 50). MUC4 is similar to MUC1 in that it is a membrane-bound mucin that has cytoplasmic domains that may mediate signal transduction (30, 77, 78). The structures of MUC1 and MUC4 have some differences, and diagrams of the domains of MUC1 and MUC4 are shown in **Figure 5**. Each mucin contains a heavily O-glycosylated extracellular domain, a transmembrane spanning (TM) domain that anchors the mucin to the cellular surface and a cytoplasmic tail (CT). Each extracellular domain contains a tandem repeat (TR) region, which is also heavily O-glycosylated with a variable number of amino acid repeats. MUC1 has 20-125 repeats of a 20 amino acid sequence and MUC4 has 145-395 repeats of a 16 amino acid sequence. Each extracellular domain may also contain an AFP binding site. Both of MUC1 and MUC4 have proteolytic cleavage sites. MUC1 could be cut by the proteolytic cleavage sites in the N-glycosylated SEA (sperm protein, enterokinase and agrin) domains. However, MUC4 is thought to be cleaved at a GDPH proteolytic site in the EGF domain which leads to generate two subunits: MUC4a and MUC4b. MUC4a is a mucin-like, heavily glycosylated subunit that can be soluble, while MUC4b is a membrane-anchored growth factor-like subunit due to the presence of EGF-like domains that are unique to that mucin (30, 45).

**Figure 6:**
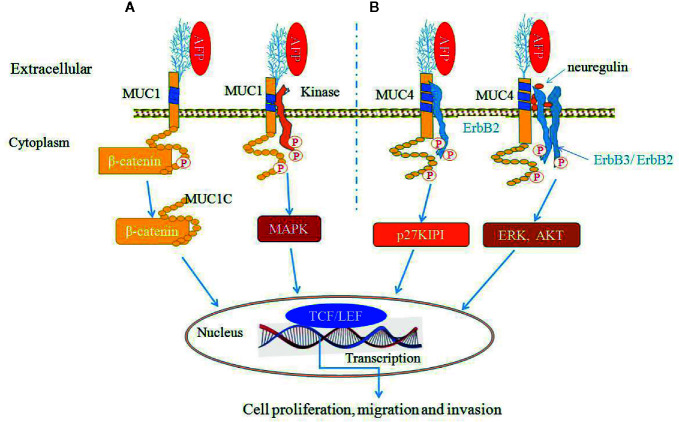
Alpha-fetoprotein (AFP) can bind to mucin 1 (MUC1) and MUC4 and synergistically promote cancer cells growth (9, 30, 34, 50). **(A)** AFP binding to MUC1 can activate MUC1 and affect β-catenin signal transduction by cleaving MUC1C in the cytoplasm and translocating β-catenin to the nucleus, thereby influencing transcription through TCF/LEF and/or other transcription factors. MUC1C can also prevent β-catenin degradation. Also, AFP can phosphorylate MUC1 and activate mitogen-activated protein kinase (MAPK) signaling. **(B)** AFP binds to MUC4, which promotes MUC4 interactions with the ErbB2 tyrosine-kinase receptor and leads to the activation of KIP1 (also known as p27). AFP can also enhance MUC4 interactions with ErbB2, ErbB3, and neuregulin, resulting in enhancing cancer cells proliferation and apoptosis inhibition through activation of the ERK and Akt signaling pathways.

AFP can bind to MUC1 and MUC4 to synergistically promote cancer cells proliferation, migration and invasion (9, 30, 34, 50) (**Figure 6**). MUC4 is intramembranous and can interact with the receptor tyrosine kinase erythroblastic oncogene B2 (ErbB2), which is related to the regulation of p27, a cyclin-dependent kinase inhibitor that is involved in the control of the G1 passage to S phases of the cell cycle. AFP binding to MUC4 can promote cell proliferation synergistically with ErbB2/B3 and neuregulin (74, 79). Mucin receptor occupancy by AFP and activation of ErbB2/B3 can upregulate the cell cycle suppressor p27 KIP, resulting in cell cycle arrest at the G1/S transition point (9, 30). The overexpression of p27 KIP in the G1 phase can prevent subsequent mitosis and lead to cell proliferation (9, 30). AFP binding to ErbB2 can also enhance MUC4 interactions with ErbB2, ErbB3, and neuregulin, increase the phosphorylation of the ErbB2 complex and result in enhancing cancer cell proliferation, migration, invasion and inhibition of apoptosis through activating ERK and AKT signaling pathways (74, 78, 79) (**Figure 6**).

Documents have indicated that the function of MUC4 is similar to that of AFPR (9, 30, 34). AFP is a growth-promoting factor, and AFP binds to AFPRs, resulting in cell proliferation, metastasis, apoptosis inhibition and immunosuppression. Overexpression of MUC4 not only can promote tumor cells proliferation and invasion, but also protect cells from lymphokine attack. Additionally, MUC4 has been shown to reduce the interactions between cells, promote cell separation, and promote tumor metastasis (30, 50). AFPR was reported to phosphorylate a 185-kDa ErbB2 protein (30), while MUC4 can bind to 185-kDa ErbB2 and induce phosphorylation of ErbB2 at the 1139Y and 1248Y sites, which results in activity of AKT and its related signaling pathways leading to promotion of cell survival (30, 80–82). MUC4 is highly expressed in breast cancer, and AFPRs were first isolated from the human MCF-7 breast cancer cell line (30). Additionally, both MUC4 and AFPRs react with peanut agglutinin lectin, and both molecules are present on many cells derived from human carcinomas, T cell lymphomas, macrophages, etc. Both receptor proteins are highly glycosylated with multiple O-linked glycans. Therefore, MUC4 may also be a viable candidate for an AFP receptor (9, 30, 82, 83).

## Scavenger Receptors (SRs)

**Figure 7:**
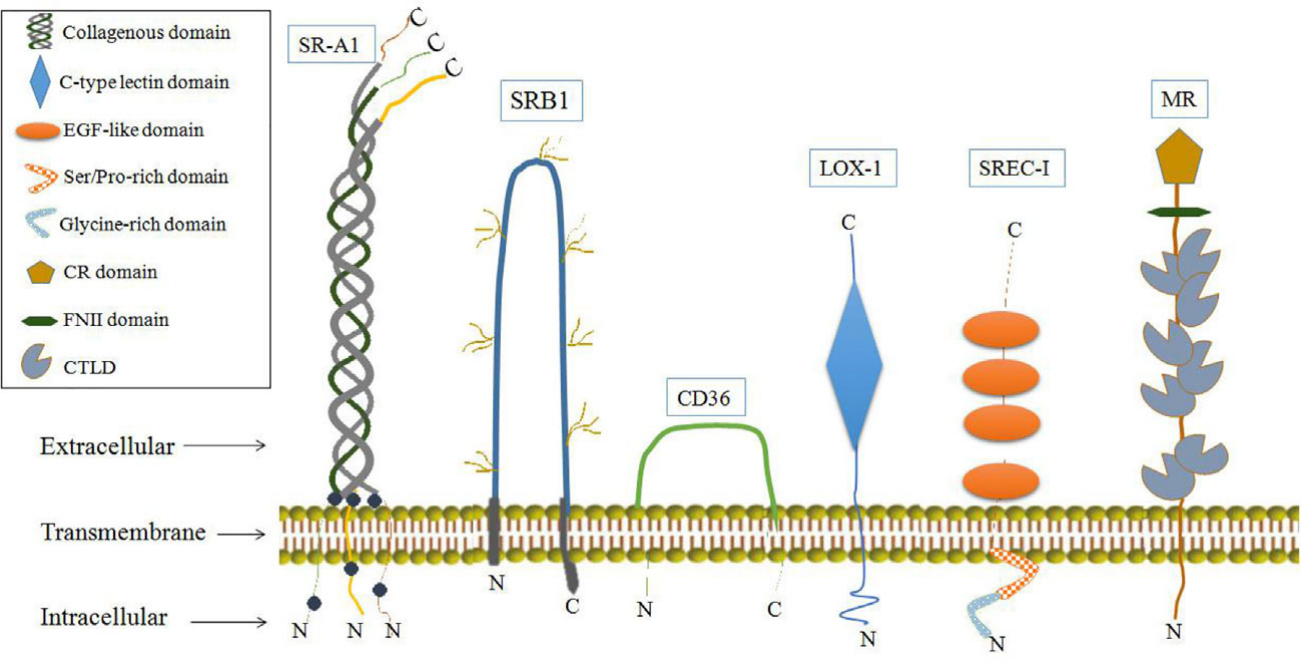
The location and structure of scavenger receptors.

It has also been shown that some SRs may function as AFPRs. SRs are single or double transmembrane-spanning glycoproteins that bind a wide variety of ligands, including modified or oxidized low-density lipoproteins, apoptotic cells and pathogens. SRs can be broadly classified into at least eight classes (A-H) (84, 85). Some SRs have biological effects involving physiological activities associated with AFP binding, and these SRs have been detected in cells or tissues which involved the uptake and cytoplasmic trafficking of AFP and proposed as AFPRs (29, 33, 34). SRs that may serve as AFPRs are shown in **Figure 7**. Among them, LOX-1 and mannose receptors have the most biological activities, similar to those of AFPRs (29, 86).

LOX-1 is a class E scavenger receptor, which has a single lectin-like gene product and is also called lectin-like oxidized low-density lipoprotein receptor 1 (87). LOX-1 is primarily expressed in endothelial cells, cardiomyocytes, smooth muscle cells, B cells, macrophages, Dendritic cells (DCs), and platelets (87). LOX-1 has multiple functions involved in lipid metabolism, cholesterol biosynthesis, and atherogenesis (88, 89). Recent studies identified LOX-1 as a possible link between obesity, dyslipidemia, and cancer (90, 91). In mouse xenograft models, LOX-1 was shown to be highly expressed in patient specimens with late-stage metastatic breast cancer and prostate cancer (90). LOX-1 knockdown or blockade in transformed cells impaired anchorage-independent growth, cell migration and invasion (90, 91).

**Figure 8:**
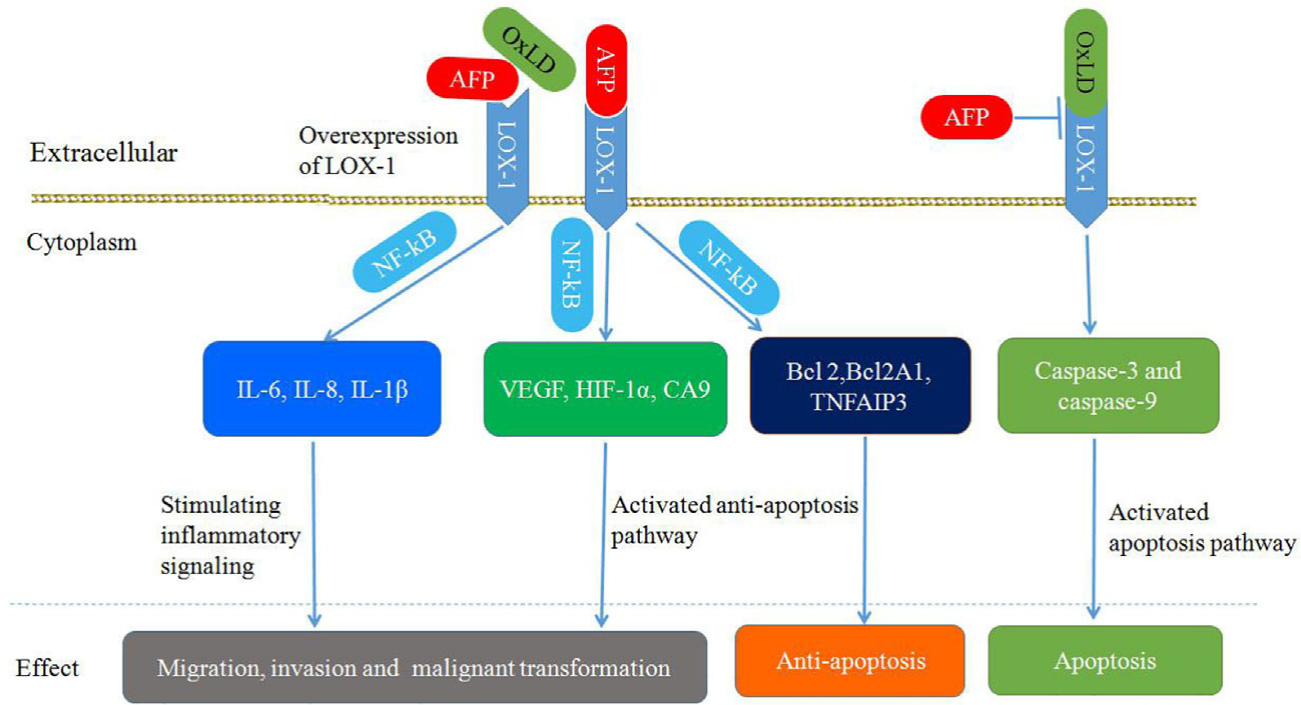
Schematic diagram of Alpha-fetoprotein (AFP) binding to LOX-1, activating signal transduction pathways and mediating migration, invasion, malignant transformation, and apoptosis inhibition in cancer cells.

As shown in **Figure 8**, the upregulation of LOX-1 stimulates inflammatory signaling (IL-6, IL-8, and IL-1β) and hypoxia-regulated pathways (VEGF, HIF-1α, and carbonic anhydrase 9) in an NF-κB-dependent manner, leading to cellular transformation and maintenance of the transformed state. Overexpression of LOX1 activates an NF-κB-dependent antiapoptotic pathway (BCL2, BCL2A1, and TNFAIP3) (85). Upregulation of endothelial LOX-1 by TNF-α facilitates the adhesion and transendothelial migration of breast cancer cells (85, 91, 92). OxLDL binding to LOX-1 can induce apoptosis by enhancing caspase-3 and caspase-9 activity; however, AFP binding to LOX-1 can inhibit OxLDL binding, which blocks the apoptosis pathway (86, 93). Additionally, LOX-1 functions as a receptor for Hsp60 and Hsp70 on mouse or human DCs. Anti-LOX-1 neutralizing antibodies can inhibit Hsp70 binding to DCs, which results in trigger the antigen cross-presentation, as well as a subsequent antigen-specific CD8^+^ T cell response and initiation of antitumor immunity (85, 94, 95).

The function of LOX-1 described above is similar to the function of AFPR. Regarding tumor growth regulation by AFP, LOX-1 has been reported to be expressed mainly on DCs, hepatoma cells, breast and ovarian adenocarcinomas, lymphomas, and prostate tumors to promote tumor growth (29, 34). AFP has been reported to be involved in transplacental passage, and LOX-1 has been localized on the membrane surface of various types of placental cells. The effect of AFP on fetal osteoporosis could likewise be linked to LOX-1 receptor. Therefore, LOX-1 may be a candidate cell-surface receptor for AFP (30, 34).

SR-A1 may also be a cell-surface receptor for AFP which are belong to class SRs-A, whose isoforms are largely expressed on macrophages but can also be detected on endothelial and smooth muscle tissues (29, 85). SR-A1 can cause the tyrosine phosphorylation of phosphatidylinositol 3-kinase (PI3-kinase), and SR-A1 activates signaling pathways involving protein kinase C (PKC), ERK, JNK, caspases and cytokine secretion (85, 86, 96–98). Ligand binding to SR-A1 can also induce tumor cell growth. These characteristics further support SR-A1 as a cell-surface receptor for AFP (29, 85, 86, 96–98). Another SR that may be candidate as an AFPR is the mannose receptor (CD206, or MR), which plays an important role in the immune response (99, 100). The MRs are endocytic receptors that share a similar structure of its family in consisting of an N-terminal CR domain, a FNII, and 8 CTLDs (**Figure 7**) (101, 102). MRs can endocytose and traffic AFP within human dendritic cells (DCs); then, they release AFP to the perinuclear region and the receptor is re-cycled back to the cell membrane. By these pathways, AFP is available to be processed within the histocompatibility complex -class II (MHC- II) system leading to immunologic responses that induce antigen-specific CD4^+^ and CD8^+^ T cell activation (33, 100). In liver cancer cells (HepG2), they more like employ CD36, and a lesser degree the LOX-1, SRB1 and SREC-1 (**Figure 7**) scavenger receptors rather than mannose receptor for uptake of AFP. These SRs can form cooperative complexes with the Toll-like receptor-2 (TRL2) results in enhancing endocytosis of AFP, receptor signaling and cross-presentation of antigens for T cell activation (33, 100).

## Conclusions and Future Perspectives

AFP binds to its receptors and regulates tumorigenesis, cancer invasion, and the antitumor immune response. Due to their complex types, polymer structure and carbohydrate composition, AFPRs have not yet been clearly identified, but according to reports of their functions in tumors, AFPRs may belong to the G protein-coupled receptors (GPCRs), mucin (MUC) receptor family, scavenger receptor (SR) families and other cell surface receptors. We have focused on two candidate receptor protein family termed the MUC receptor family and SR family. In the MUC receptor family, MUC1 and MUC4 are proposed to be AFPRs. Among the scavenger receptors, LOX-1 and CD206, SR-A1, CD36, SRB1 and SREC-1 are proposed to be AFPRs. MUC1 is now being used to develop cancer vaccines for several malignant tumors, including breast, prostate, pancreas, and colon (56, 69, 103) and will be utilized in human clinical trials in the next decade or more (7, 9). Other AFP candidate receptor proteins will also be utilized in vaccine design. For example, LOX-1 vaccines may be used to enhance the T cell response and tumor eradication (29, 94). SR-A1 vaccines will also be utilized in vaccination-induced T cell activation and antitumor immune response, and SR-A1 blockade can improve the antitumor efficacy of DCs vaccines (85). Although AFPRs or AFP vaccines have not been used in clinic, the association between them and high tumor growth rate and poor clinical outcome in HCC (27, 29–33) supports AFPRs as an immunogenic tumor-associated antigen target; as such delivered drug have been designed to target AFPRs to eliminate cancer cells (7–9, 104, 105). Additionally, recent study found that scavenger receptor (MR) could process AFP leading to antigen presentation to T-lymphocytes at the dendritic cell surface (33, 100). So developing vaccines or drugs through AFPRs will be used to verify validity and practicability for clinic trials in the future.

In addition, other types of receptors or binding proteins may be proposed as AFPRs that have been reported in the literature. With the development of bio-technology, AFPRs may be used to verify the validity in the future by the following procedures: 1) binding affinity analysis, 2) immunofluorescence and/or radiolabeled AFP (visual) binding to cell membranes, 3) “in silico” computer modeling, or 4) Fluorescence resonance energy transfer (FRET) analysis (30, 34). A better understanding of AFPRs will facilitate the development of rational approaches for drug delivery, antagonizing immune suppression and diagnostic imaging, which will lead to improved outcomes in cancer treatment. For example, AFPRs may be utilized to facilitate the design of AFP-derived peptides to target functionally distinct receptors and prevent cancer growth and progression (7–9, 104, 105). Overall, the AFP binding proteins could be utilized in vaccine designs, or used as a bio-targets to precisely design AFP-derived peptides conjugated to toxins that selectively bind to the receptors in order to serve as targeting agents for treatment of cancers.

## Author Contributions 

BL, QW, and KL gathered the related literature, prepared the figures and drafted the manuscript. XD, MZ, and ML participated in the design of the review and drafted the manuscript. All authors contributed to the article and approved the submitted version.

## Funding

This work was supported by the National Natural Science Foundation of China (Nos. 82060514, 81960519, 81660463, 81560450, and 31560243), The Natural Science Foundation of Hainan Province (Nos. 820RC634, 2019CXTD406, 2019CR204, and 20168263), Hainan Provincial Association for Science and Technology Program of Youth Science Talent and Academic Innovation (No. QCXM 201922).

## Conflict of Interest

The authors declare that the research was conducted in the absence of any commercial or financial relationships that could be construed as a potential conflict of interest.

## Abbreviations

AFP, Alpha-fetoprotein; AFPR, AFP receptor; ATRA, all-trans retinoic acid; cAMP, cyclic adenosine 3’, 5’-monophosphate; CR, cysteine-rich domain; CTLD, C-type lectin-like domain, CXCR4, C-X-C chemokine receptor type 4; EGF, epidermal growth factor; FNII, fibronectin type II domain; GPCRs, G protein-coupled receptors; HCC, hepatocellular carcinoma; MAPK, mitogen-activated protein kinase; PTEN, phosphatase and tensin homolog, gene deleted on chromosome 10; RAR, retinoic acid receptor; TR, tandem repeat; TRAIL, tumor necrosis-factor related apoptosis-induced ligand.

## References

[1] Bei R Mizejewski GJ. Alpha-fetoprotein is an autoantigen in hepatocellular carcinoma and juvenile Batten disease. Front Biosci (Landmark Ed) (2020) 25:912–29. 10.2741/4840 31585923

[2] Kim H Lee SJ Yoon M. Alpha-fetoprotein is correlated with intrahepatic recurrence of hepatocellular carcinoma after a hepatectomy. Ann Surg Treat Res (2020) 98:168–76. 10.4174/astr.2020.98.4.168 PMC7118319 32274364

[3] Mizejewski GJ. Alpha-fetoprotein structure and function: relevance to isoforms, epitopes, and conformational variants. Exp Biol Med (Maywood) (2001) 226:377–408. 10.1177/153537020122600503 11393167

[4] Bai DS Zhang C Chen P Jin SJ Jiang GQ. The prognostic correlation of AFP level at diagnosis with pathological grade, progression, and survival of patients with hepatocellular carcinoma. Sci Rep (2017) 7:12870. 10.1038/s41598-017-12834-1 28993684 PMC5634482

[5] Mehta N Dodge JL Grab JD Yao FY. National Experience on Down-Staging of Hepatocellular Carcinoma Before Liver Transplant: Influence of Tumor Burden, Alpha-Fetoprotein, and Wait Time. Hepatology (2020) 71:943–54. 10.1002/hep.30879 PMC8722406 31344273

[6] EASL Clinical Practice Guidelines: Management of hepatocellular carcinoma. J Hepatol (2018) 69(1):182–236. 10.1016/j.jhep.2018.03.019 29628281

[7] Pak VN. The use of alpha-fetoprotein for the treatment of autoimmune diseases and cancer. Ther Deliv (2018) 9:37–46. 10.4155/tde-2017-0073 29216804

[8] Pak V. The use of α-fetoprotein for the delivery of cytotoxic payloads to cancer cells. Ther Deliv (2014) 5:885–92. 10.4155/tde.14.59 25337646

[9] Mizejewski GJ. The adenocarcinoma cell surface mucin receptor for alpha-fetoprotein: is the same receptor present on circulating monocytes and macrophages? A commentary. Tumour Biol (2014) 35:7397–402. 10.1007/s13277-014-2183-7 24916573

[10] Li MS Li PF He SP Du GG Li G. The promoting molecular mechanism of alpha-fetoprotein on the growth of human hepatoma Bel7402 cell line. World J Gastroenterol (2002) 8:469–75. 10.3748/wjg.v8.i3.469 PMC4656423 12046072

[11] Li MS Li PF Yang FY He SP Du GG Li G. The intracellular mechanism of alpha-fetoprotein promoting the proliferation of NIH 3T3 cells. Cell Res (2002) 12:151–6. 10.1038/sj.cr.7290121 12118941

[12] Zheng Y Zhu M Li M. Effects of alpha-fetoprotein on the occurrence and progression of hepatocellular carcinoma. J Cancer Res Clin Oncol (2020) 146:2439–46. 10.1007/s00432-020-03331-6 PMC11804406 32725355

[13] Xue J Cao Z Cheng Y Wang J Liu Y Yang R. Acetylation of alpha-fetoprotein promotes hepatocellular carcinoma progression. Cancer Lett (2020) 471:12–26. 10.1016/j.canlet.2019.11.043 31811908

[14] Wang S Zhu M Wang Q Hou Y Li L Weng H. Alpha-fetoprotein inhibits autophagy to promote malignant behaviour in hepatocellular carcinoma cells by activating PI3K/AKT/mTOR signalling. Cell Death Dis (2018) 9:1027. 10.1038/s41419-018-1036-5 30301886 PMC6177398

[15] Li M Li H Li C Wang S Jiang W Liu Z. Alpha-fetoprotein: a new member of intracellular signal molecules in regulation of the PI3K/AKT signaling in human hepatoma cell lines. Int J Cancer (2011) 128:524–32. 10.1002/ijc.25373 20473866

[16] Wang FZ Fei HR Lian LH Wang JM Qiu YY. Hepatitis B x-interacting protein induces HepG2 cell proliferation through activation of the phosphatidylinositol 3-kinase/Akt pathway. Exp Biol Med (Maywood) (2011) 236:62–9. 10.1258/ebm.2010.010179 21239735

[17] Missiaglia E Dalai I Barbi S Beghelli S Falconi M della Peruta M. Pancreatic endocrine tumors: expression profiling evidences a role for AKT-mTOR pathway. J Clin Oncol (2010) 28:245–55. 10.1200/JCO.2008.21.5988 PMC4288616 19917848

[18] Yang X Chen L Liang Y Si R Jiang Z Ma B. Knockdown of alpha-fetoprotein expression inhibits HepG2 cell growth and induces apoptosis. J Cancer Res Ther (2018) 14:S634–43. 10.4103/0973-1482.180681 30249880

[19] Zhu M Li W Lu Y Dong X Chen Y Lin B. Alpha fetoprotein antagonizes apoptosis induced by paclitaxel in hepatoma cells in vitro. Sci Rep (2016) 6:26472. 10.1038/srep26472 27255186 PMC4891737

[20] Li M Li H Li C Zhou S Guo L Liu H. Alpha fetoprotein is a novel protein-binding partner for caspase-3 and blocks the apoptotic signaling pathway in human hepatoma cells. Int J Cancer (2009) 124:2845–54. 10.1002/ijc.24272 19267404

[21] Yang X Zhang Y Zhang L Zhang L Mao J. Silencing alpha-fetoprotein expression induces growth arrest and apoptosis in human hepatocellular cancer cell. Cancer Lett (2008) 271:281–93. 10.1016/j.canlet.2008.06.017 18657899

[22] Li M Li H Li C Guo L Liu H Zhou S. Cytoplasmic alpha-fetoprotein functions as a co-repressor in RA-RAR signaling to promote the growth of human hepatoma Bel 7402 cells. Cancer Lett (2009) 285:190–9. 10.1016/j.canlet.2009.05.014 19501957

[23] Li M Zhou S Liu X Li P McNutt MA Li G. alpha-Fetoprotein shields hepatocellular carcinoma cells from apoptosis induced by tumor necrosis factor-related apoptosis-inducing ligand. Cancer Lett (2007) 249:227–34. 10.1016/j.canlet.2006.09.004 17046153

[24] Zhang C Zhang J Wang J Yan Y Zhang C. Alpha-fetoprotein accelerates the progression of hepatocellular carcinoma by promoting Bcl-2 gene expression through an RA-RAR signalling pathway. J Cell Mol Med (2020) 24:13804–12. 10.1111/jcmm.15962 PMC7753843 33090723

[25] Helmy SA El-Mesery M El-Karef A Eissa LA. El Gayar AM.Thymoquinone upregulates TRAIL/TRAILR2 expression and attenuates hepatocellular carcinoma in vivo model. Life Sci (2019) 15(233):116673. 10.1016/j.lfs.2019.116673 31336121

[26] Meng W Li X Bai Z Li Y Yuan J Liu T. Silencing alpha-fetoprotein inhibits VEGF and MMP-2/9 production in human hepatocellular carcinoma cell. PloS One (2014) 9:e90660. 10.1371/journal.pone.0090660 24587407 PMC3938808

[27] Yamashita T Forgues M Wang W Kim JW Ye Q Jia H. EpCAM and alpha-fetoprotein expression defines novel prognostic subtypes of hepatocellular carcinoma. Cancer Res (2008) 68:1451–61. 10.1158/0008-5472.CAN-07-6013 18316609

[28] Shan YF Huang YL Xie YK Tan YH Chen BC Zhou MT. Angiogenesis and clinicopathologic characteristics in different hepatocellular carcinoma subtypes defined by EpCAM and α-fetoprotein expression status. Med Oncol (2011) 28:1012–6. 10.1007/s12032-010-9600-6 20571936

[29] Mizejewski GJ. Review of the putative cell-surface receptors for alpha-fetoprotein: identification of a candidate receptor protein family. Tumour Biol (2011) 32:241–58. 10.1007/s13277-010-0134-5 21120646

[30] Mizejewski GJ. Review of the adenocarcinoma cell surface receptor for human alpha-fetoprotein; proposed identification of a widespread mucin as the tumor cell receptor. Tumour Biol (2013) 34:1317–36. 10.1007/s13277-013-0704-4 23446764

[31] Geuskens M Naval J Uriel J. Ultrastructural studies of the intracellular translocation of endocytosed alpha-foetoprotein (AFP) by cytochemistry and of the uptake of 3H-arachidonic acid bound to AFP by autoradiography in rat rhabdomyosarcoma cells. J Cell Physiol (1986) 128:389–96. 10.1002/jcp.1041280307 2427529

[32] Torres JM Geuskens M Uriel J. Receptor-mediated endocytosis and recycling of alpha-fetoprotein in human B-lymphoma and T-leukemia cells. Int J Cancer (1991) 47:110–7. 10.1002/ijc.2910470120 1702404

[33] Mizejewski GJ. Alpha-fetoprotein uptake and cytoplasmic trafficking in cancer and immune-associated cells: Relevance to adaptive immunity. EC Clin Exp Anatomy (2018) 1:71–7.

[34] Mizejewski GJ. Protein binding and interactions with alpha-fetoprotein(AFP): A review of multiple AFP cell surface receptors, intracytoplasmic binding, and inter- molecular complexing proteins. J Mol Cell Biol Forecast (2019) 2:1016.

[35] Laderoute M Willans D Wegmann T Longenecker M. The identification, isolation and characterization of a 67 kilodalton, PNA-reactive autoantigen commonly expressed in human adenocarcinomas. Anticancer Res (1994) 14:1233–45. 7520680

[36] Moser B Wolf M Walz A Loetscher P. Chemokines: multiple levels of leukocyte migration control. Trends Immunol (2004) 25:75–84. 10.1016/j.it.2003.12.005 15102366

[37] Atemezem A Mbemba E Marfaing R Vaysse J Pontet M Saffar L. Human alpha-fetoprotein binds to primary macrophages. Biochem Biophys Res Commun (2002) 296:507–14. 10.1016/s0006-291x(02)00909-9 12176010

[38] Atemezem A Mbemba E Vassy R Slimani H Saffar L Gattegno L. Human alpha1-acid glycoprotein binds to CCR5 expressed on the plasma membrane of human primary macrophages. Biochem J (2001) 356:121–8. 10.1042/0264-6021:3560121 PMC1221819 11336643

[39] Mizejewski GJ. Alpha-fetoprotein as a biomarker in immunodeficiencydiseases: relevance to Ataxia telangiectasia and related disorders. J Immunodefic Disor (2014) 3:1–12. 10.4172/2324-853X.1000108

[40] Mizejewski GJ. The third domain fragment of alpha-fetoprotein (AFP): Mapping AFP interaction sites with selective and non-selective cationchannels. Curr Topics Pept Protein Res (2016) 16:63–82.

[41] Zitt C Halaszovich CR Lückhoff A. The TRP family of cation channels: probing and advancing the concepts on receptor-activated calcium entry. Prog Neurobiol (2002) 66:243–64. 10.1016/s0301-0082(02)00002-3 11960680

[42] Kubo Y Adelman JP Clapham DE Jan LY Karschin A Kurachi Y. International Union of Pharmacology. LIV. Nomenclature and molecular relationships of inwardly rectifying potassium channels. Pharmacol Rev (2005) 57:509–26. 10.1124/pr.57.4.11 16382105

[43] Ashen MD O’Rourke B Kluge KA Johns DC Tomaselli GF. Inward rectifier K+ channel from human heart and brain: cloning and stable expression in ahuman cell line. Am J Physiol (1995) 268:H506–11. 10.1152/ajpheart.1995.268.1.H506 7840300

[44] Mizejewski GJ. Breast cancer and transient receptor potential (TRP) cation channels: Is there a role for non-selective TRP channels as therapeutic cancer targets? Int J Cancer Res Dev (2017) 2:4–7.

[45] Dhanisha SS Guruvayoorappan C Drishya S Abeesh P. Mucins: Structural diversity, biosynthesis, its role in pathogenesis and as possible therapeutic targets. Crit Rev Oncol Hematol (2018) 122:98–122. 10.1016/j.critrevonc.2017.12.006 29458795

[46] Seregni E Botti C Massaron S Lombardo C Capobianco A Bogni A. Structure, function and gene expression of epithelial mucins. Tumori (1997) 83:625–32. 10.1177/030089169708300301 9267478

[47] Qiu SM Wen G Wen J Soloway RD Crowther RS. Interaction of human gallbladder mucin with calcium hydroxyapatite: binding studies and the effect on hydroxyapatite formation. Hepatology (1995) 21:1618–24. 10.1002/hep.1840210621 7768507

[48] Belisle JA Horibata S Jennifer GA Petrie S Kapur A André S. Identification of Siglec-9 as the receptor for MUC16 on human NK cells, B cells, and monocytes. Mol Cancer (2010) 9:118. 10.1186/1476-4598-9-118 20497550 PMC2890604

[49] Singh PK Hollingsworth MA. Cell surface-associated mucins in signal transduction. Trends Cell Biol (2006) 16:467–76. 10.1016/j.tcb.2006.07.006 16904320

[50] Hollingsworth MA Swanson BJ. Mucins in cancer: protection and control of the cell surface. Nat Rev Cancer (2004) 4:45–60. 10.1038/nrc1251 14681689

[51] Yasumizu Y Rajabi H Jin C Hata T Pitroda S Long MD. MUC1-C regulates lineage plasticity driving progression to neuroendocrine prostate cancer. Nat Commun (2020) 11:338. 10.1038/s41467-019-14219-6 31953400 PMC6969104

[52] van Putten JPM Strijbis K. Transmembrane Mucins: Signaling Receptors at the Intersection of Inflammation and Cancer. J Innate Immun (2017) 9:281–99. 10.1159/000453594 PMC5516414 28052300

[53] Bhatia R Gautam SK Cannon A Thompson C Hall BR Aithal A. Cancer-associated mucins: role in immune modulation and metastasis. Cancer Metastasis Rev (2019) 38:223–36. 10.1007/s10555-018-09775-0 PMC6614013 30618016

[54] Rajabi H Kufe D. MUC1-C Oncoprotein Integrates a Program of EMT, Epigenetic Reprogramming and Immune Evasion in Human Carcinomas. Biochim Biophys Acta Rev Cancer (2017) 1868:117–22. 10.1016/j.bbcan.2017.03.003 PMC5548633 28302417

[55] Sheng YH Hasnain SZ Florin TH McGuckin MA. Mucins in inflammatory bowel diseases and colorectal cancer. J Gastroenterol Hepatol (2012) 27:28–38. 10.1111/j.1440-1746.2011.06909.x 21913981

[56] Nath S Mukherjee P. MUC1: a multifaceted oncoprotein with a key role in cancer progression. Trends Mol Med (2014) 20:332–42. 10.1016/j.molmed.2014.02.007 PMC5500204 24667139

[57] Bose M Mukherjee P. Microbe-MUC1 Crosstalk in Cancer-Associated Infections. Trends Mol Med (2020) 26:324–36. 10.1016/j.molmed.2019.10.003 31753595

[58] Danese E Ruzzenente A Montagnana M Lievens PM. Current and future roles of mucins in cholangiocarcinoma-recent evidences for a possible interplay with bile acids. Ann Transl Med (2018) 6:333. 10.21037/atm.2018.07.16 30306072 PMC6174188

[59] Singh R Bandyopadhyay D. MUC1: a target molecule for cancer therapy. Cancer Biol Ther (2007) 6:481–6. 10.4161/cbt.6.4.4201 18027437

[60] Zhou D Xu L Huang W Tonn T. Epitopes of MUC1 tandem repeats in cancer as revealed by antibody crystallography: Toward glycopeptide signature-guided therapy. Molecules (2018) 23:1326. 10.3390/molecules23061326 PMC6099590 29857542

[61] Kumagai A Kondo F Sano K Inoue M Fujii T Hashimoto M. Immunohistochemical study of hepatocyte, cholangiocyte and stem cell markers of hepatocellular carcinoma: the second report: relationship with tumor size and cell differentiation. J Hepatobiliary Pancreat Sci (2016) 23:414–21. 10.1002/jhbp.356 PMC5029768 27161394

[62] Bozkaya G Korhan P Cokaklı M Erdal E Sağol O Karademir S. Cooperative interaction of MUC1 with the HGF/c-Met pathway during hepatocarcinogenesis. Mol Cancer (2012) 11:64. 10.1186/1476-4598-11-64 22962849 PMC3542123

[63] Li Q Wang F Liu G Yuan H Chen T Wang J. Impact of Mucin1 knockdown on the phenotypic characteristics of the human hepatocellular carcinoma cell line SMMC-7721. Oncol Rep (2014) 31:2811–9. 10.3892/or.2014.3136 24737121

[64] Li Q Liu G Shao D Wang J Yuan H Chen T. Mucin1 mediates autocrine transforming growth factor beta signaling through activating the c-Jun N-terminal kinase/activator protein 1 pathway in human hepatocellular carcinoma cells. Int J Biochem Cell Biol (2015) 59:116–25. 10.1016/j.biocel.2014.11.012 25526895

[65] Li Q Liu G Yuan H Wang J Guo Y Chen T. Mucin1 shifts Smad3 signaling from the tumor-suppressive pSmad3C/p21(WAF1) pathway to the oncogenic pSmad3L/c-Myc pathway by activating JNK in human hepatocellular carcinoma cells. Oncotarget (2015) 6:4253–65. 10.18632/oncotarget.2973 PMC4414187 25714018

[66] Wang J Liu G Li Q Wang F Xie F Zhai R. Mucin1 promotes the migration and invasion of hepatocellular carcinoma cells via JNK-mediated phosphorylation of Smad2 at the C-terminal and linker regions. Oncotarget (2015) 6:19264–78. 10.18632/oncotarget.4267 PMC4662489 26057631

[67] Wang J Ni WH Hu KB Zhai XY Xie F Jie J. Targeting MUC1 and JNK by RNA interference and inhibitor inhibit the development of hepatocellular carcinoma. Cancer Sci (2017) 108:504–11. 10.1111/cas.13144 PMC5378288 28012230

[68] Xu HL Inagaki Y Seyama Y Sugawara Y Kokudo N Nakata M. Expression of KL-6 mucin, a human MUC1 mucin, in intrahepatic cholangiocarcinoma and its potential involvement in tumor cell adhesion and invasion. Life Sci (2009) 85:395–400. 10.1016/j.lfs.2009.07.004 19631667

[69] Nabavinia MS Gholoobi A Charbgoo F Nabavinia M Ramezani M Abnous K. Anti-MUC1 aptamer: A potential opportunity for cancer treatment. Med Res Rev (2017) 37:1518–39. 10.1002/med.21462 28759115

[70] Farahmand L Merikhian P Jalili N Darvishi B. Majidzadeh-A K. Significant Role of MUC1 in Development of Resistance to Currently Existing Anti-cancer Therapeutic Agents. Curr Cancer Drug Targets (2018) 18:737–48. 10.2174/1568009617666170623113520 28669345

[71] Ren J Raina D Chen W Li G Huang L Kufe D. MUC1 oncoprotein functions in activation of fibroblast growth factor receptor signaling. Mol Cancer Res (2006) 4:873–83. 10.1158/1541-7786.MCR-06-0204 PMC3322466 17114345

[72] Schroeder JA Adriance MC Thompson MC Camenisch TD Gendler SJ. MUC1 alters beta-catenin-dependent tumor formation and promotes cellular invasion. Oncogene (2003) 22:1324–32. 10.1038/sj.onc.1206291 12618757

[73] Wei X Xu H Kufe D. MUC1 oncoprotein stabilizes and activates estrogen receptor alpha. Mol Cell (2006) 21:295–305. 10.1016/j.molcel.2005.11.030 16427018

[74] Kasprzak A Adamek A. Mucins: the Old, the new and the promising factors in hepatobiliary carcinogenesis. Int J Mol Sci (2019) 20:1288. 10.3390/ijms20061288 PMC6471604 30875782

[75] Yi FT Lu QP. Mucin 1 promotes radioresistance in hepatocellular carcinoma cells through activation of JAK2/STAT3 signaling. Oncol Lett (2017) 14:7571–6. 10.3892/ol.2017.7119 PMC5755255 29344203

[76] Agata N Ahmad R Kawano T Raina D Kharbanda S Kufe D. MUC1 oncoprotein blocks death receptor-mediated apoptosis by inhibiting recruitment of caspase-8. Cancer Res (2008) 68:6136–44. 10.1158/0008-5472.CAN-08-0464 PMC2536759 18676836

[77] Byrd JC Bresalier RS. Mucins and mucin binding proteins in colorectal cancer. Cancer Metastasis Rev (2004) 23:77–99. 10.1023/a:1025815113599 15000151

[78] Mercogliano MF De Martino M Venturutti L Rivas MA Proietti CJ Inurrigarro G. TNFα-induced Mucin 4 expression elicits trastuzumab resistance in HER2-positive breast cancer. Clin Cancer Res (2017) 23:636–48. 10.1158/1078-0432.CCR-16-0970 27698002

[79] Jepson S Komatsu M Haq B Arango ME Huang D Carraway CA. Muc4/sialomucin complex, the intramembrane ErbB2 ligand, induces specific phosphorylation of ErbB2 and enhances expression of p27(kip), but does not activate mitogen-activated kinase or protein kinaseB/Akt pathways. Oncogene (2002) 21:7524–32. 10.1038/sj.onc.1205970 12386815

[80] Ramsauer VP Pino V Farooq A Carothers Carraway CA Salas PJ Carraway KL. Muc4-ErbB2 complex formation and signaling in polarized CACO-2 epithelial cells indicate that Muc4 acts as an unorthodox ligand for ErbB2. Mol Biol Cell (2006) 17:2931–41. 10.1091/mbc.e05-09-0895 PMC1483030 16624867

[81] Horowitz JC Lee DY Waghray M Keshamouni VG Thomas PE Zhang H. Activation of the pro-survival phosphatidylinositol 3-kinase/AKT pathway by transforming growth factor-beta1 in mesenchymal cells is mediated by p38 MAPK-dependent induction of an autocrine growth factor. J Biol Chem (2004) 279:1359–67. 10.1074/jbc.M306248200 PMC1360222 14576166

[82] Bae JS Lee J Park Y Park K Kim JR Cho DH. Attenuation of MUC4 potentiates the anticancer activity of auranofin via regulation of the Her2/Akt/FOXO3 pathway in ovarian cancer cells. Oncol Rep (2017) 38:2417–25. 10.3892/or.2017.5853 28765909

[83] Gautam SK Kumar S Dam V Ghersi D Jain M Batra SK. MUCIN-4 (MUC4) is a novel tumor antigen in pancreatic cancer immunotherapy. Semin Immunol (2020) 47:101391. 10.1016/j.smim.2020.101391 31952903 PMC7160012

[84] Prabhudas M Bowdish D Drickamer K Febbraio M Herz J Kobzik L. Standardizing scavenger receptor nomenclature. J Immunol (2014) 192:1997–2006. 10.4049/jimmunol.1490003 24563502 PMC4238968

[85] Yu X Guo C Fisher PB Subjeck JR Wang XY. Scavenger receptors: Emerging roles in cancer biology and immunology. Adv Cancer Res (2015) 128:309–64. 10.1016/bs.acr.2015.04.004 PMC4631385 26216637

[86] Murphy JE Tedbury PR Homer-Vanniasinkam S Walker JH Ponnambalam S. Biochemistry and cell biology of mammalian scavenger receptors. Atherosclerosis (2005) 182:1–15. 10.1016/j.atherosclerosis.2005.03.036 15904923

[87] Chen M Masaki T Sawamura T. LOX-1, the receptor for oxidized low-density lipoprotein identified from endothelial cells: implications in endothelial dysfunction and atherosclerosis. Pharmacol Ther (2002) 95:89–100. 10.1016/s0163-7258(02)00236-x 12163130

[88] Huysamen C Brown GD. The fungal pattern recognition receptor, Dectin-1, and the associated cluster of C-type lectin-like receptors. FEMS Microbiol Lett (2009) 290:121–8. 10.1111/j.1574-6968.2008.01418.x PMC2704933 19025564

[89] Mehta JL Sanada N Hu CP Chen J Dandapat A Sugawara F. Deletion of LOX-1 reduces atherogenesis in LDLR knockout mice fed high cholesterol diet. Circ Res (2007) 100:1634–42. 10.1161/CIRCRESAHA.107.149724 17478727

[90] Hirsch HA Iliopoulos D Joshi A Zhang Y Jaeger SA Bulyk M. A transcriptional signature and common gene networks link cancer with lipid metabolism and diverse human diseases. Cancer Cell (2010) 17:348–61. 10.1016/j.ccr.2010.01.022 PMC2854678 20385360

[91] Balzan S Lubrano V. Role of Ox-LDL and LOX-1 in Atherogenesis. Life Sci (2018) 198:79–86. 10.1016/j.lfs.2018.02.024 29462603

[92] Liang M Zhang P Fu J. Up-regulation of LOX-1 expression by TNF-alpha promotes trans-endothelial migration of MDA-MB-231 breast cancer cells. Cancer Lett (2007) 258:31–7. 10.1016/j.canlet.2007.08.003 17868983

[93] Hofmann A Brunssen C Morawietz H. Contribution of lectin-like oxidized low-density lipoprotein receptor-1 and LOX-1 modulating compounds to vascular diseases. Vascul Pharmacol (2018) 107:1–11. 10.1016/j.vph.2017.10.002 29056472

[94] Delneste Y Magistrelli G Gauchat J Haeuw J Aubry J Nakamura K. Involvement of LOX-1 in dendritic cell-mediated antigen cross-presentation. Immunity (2002) 17:353–62. 10.1016/s1074-7613(02)00388-6 12354387

[95] Murshid A Theriault J Gong J Calderwood SK. Molecular Chaperone Receptors. Methods Mol Biol (2018) 1709:331–44. 10.1007/978-1-4939-7477-1_24 PMC6777869 29177670

[96] Sapkota M DeVasure JM Kharbanda KK Wyatt TA. Malondialdehyde-acetaldehyde (MAA) adducted surfactant protein induced lung inflammation is mediated through scavenger receptor a (SR-A1). Respir Res (2017) 18:36. 10.1186/s12931-017-0517-x 28193223 PMC5307820

[97] Coller SP Paulnock DM. Signaling pathways initiated in macrophages after engagement of type A scavenger receptors. J Leukoc Biol (2001) 70:142–8. 10.1189/jlb.70.1.142 11435497

[98] Guo S Ni Y Ben J Xia Y Zhou T Wang D. Class A Scavenger Receptor Exacerbates Osteoclastogenesis by an Interleukin-6-Mediated Mechanism through ERK and JNK Signaling Pathways. Int J Biol Sci (2016) 12(10):1155–67. 10.7150/ijbs.14654 PMC5069438 27766031

[99] Jaynes JM Sable R Ronzetti M Bautista W Knotts Z Abisoye-Ogunniyan A. Mannose receptor (CD206) activation in tumor-associated macrophages enhances adaptive and innate antitumor immune responses. Sci Transl Med (2020) 12:eaax6337. 10.1126/scitranslmed.aax6337 32051227 PMC7832040

[100] Pardee AD Yano H Weinstein AM Ponce AA Ethridge AD Normolle DP. Route of antigen delivery impacts the immunostimulatory activity of dendritic cell-based vaccines for hepatocellular carcinoma. J Immunother Cancer (2015) 3:32. 10.1186/s40425-015-0077-x 26199728 PMC4509479

[101] Martinez-Pomares L. The mannose receptor. J Leukoc Biol (2012) 92:1177–86. 10.1189/jlb.0512231 22966131

[102] Khan A Mann L Papanna R Lyu MA Singh CR Olson S. Mesenchymal stem cells internalize Mycobacterium tuberculosis through scavenger receptors and restrict bacterial growth through autophagy. Sci Rep (2017) 7:15010. 10.1038/s41598-017-15290-z 29118429 PMC5678154

[103] Hossain MK Wall KA. Immunological Evaluation of Recent MUC1 Glycopeptide Cancer Vaccines. Vaccines (Basel) (2016) 4:25. 10.3390/vaccines4030025 PMC5041019 27472370

[104] Mizejewski GJ. The alpha-fetoprotein third domain receptor binding fragment: in search of scavenger and associated receptor targets. J Drug Targeting (2015) 23:538–51. 10.3109/1061186X.2015.1015538 25766080

[105] Mizejewski GJ. Mechanism of cancer growth suppression of alpha-fetoprotein derived growth inhibitory peptides (GIP): Comparison of GIP-34 versus GIP-8 (AFPep). Updates and Prospects. Cancers (Basel) (2011) 3:2709–33. 10.3390/cancers3022709 PMC3757439 24212829

